# Rapid Detection of *bla*_KPC_ in Carbapenem-Resistant *Enterobacterales* Based on CRISPR/Cas13a

**DOI:** 10.1007/s00284-023-03457-z

**Published:** 2023-09-22

**Authors:** Mingjun Liang, Bin Xiao, Lidan Chen, Xiaoyan Huang, Jinchao Li, Zhenzhan Kuang, Xinping Chen, Xiuna Huang, Zhaohui Sun, Linhai Li

**Affiliations:** 1 Department of Laboratory Medicine, General Hospital of Southern Theater Command, No. 111, Liuhua Road, Yuexiu District, Guangzhou City, 510010 Guangdong Province China; 2 Department of Laboratory Medicine, The Sixth Affiliated Hospital of Guangzhou Medical University, Qingyuan People’s Hospital, No. B24, Yinquan Road, Qingcheng District, Qingyuan City, 511518 Guangdong Province China; 3 The First School of Clinical Medicine, Southern Medical University, Guangzhou City, 510000 Guangdong Province China; 4 Department of Clinical Laboratory, Hainan Cancer Hospital, Haikou, 570311 China

## Abstract

*Klebsiella pneumoniae* carbapenemase (KPC) is a crucial enzyme that causes carbapenem resistance in *Enterobacterales*, and infections by these "superbugs" are extremely challenging to treat. Therefore, there is a pressing need for a rapid and accurate KPC detection test to control the prevalence of carbapenem-resistant *Enterobacterales* (CREs). In this study, we established a novel method for detection of *bla*_KPC_, the gene responsible for encoding KPC, based on a recombinase polymerase amplification (RPA) and a CRISPR/Cas13a reaction coupled to fluorophore activation (termed RPA-Cas13a assay). We carefully selected a pair of optimal amplification primers for *bla*_KPC_ and achieved a lower limit of detection of approximately 2.5 copies/μL by repeatedly amplifying a recombinant plasmid containing *bla*_KPC_. The RPA-Cas13a assay demonstrated a sensitivity of 96.5% and specificity of 100% when tested on 57 *bla*_KPC_-positive CRE strains, which were confirmed by DNA sequencing. Moreover, in 311 sputum samples, the theoretical antibiotic resistance characteristics of *bla*_KPC_-positive strains obtained by the RPA-Cas13a assay were highly consistent with the results of antibiotic susceptibility test (*Kappa* = 0.978 > 0.81, *P* < 0.01). In conclusion, the RPA-Cas13a system is a simple and one-hour efficient technology for the detection of a potentially fatal antibiotic resistance gene.

## Introduction

Carbapenems are currently employed as the ultimate resort for treating gram-negative bacteria, such as *Enterobacterales*. However, the escalating rate of carbapenem resistance due to widespread misuse has rendered Carbapenem-resistant *Enterobacterales* (CREs) an insurmountable adversary in clinical therapy [1]. The resistance to carbapenems in *Enterobacterales* is conferred by carbapenemases, including class A serine enzymes, class B metalloenzymes, and class D serine enzymes. Among them, *Klebsiella Pneumoniae* carbapenemase (KPC), belonging to class A, accounts for a majority of carbapenemases identified in clinical CREs isolated from China [2]. Currently, available methods for detecting carbapenemases involve phenotypic and genetic detection, accompanying with the limitations of time-consuming and demanding. Therefore, the development of rapid and accurate laboratory techniques for carbapenemase detection is crucial to control the outbreak of CRE.

Prokaryotes have evolved a clustered regularly interspaced short palindromic repeats (CRISPR)/CRISPR-associated protein (CRISPR/Cas) system to detect and destroy infecting viruses, and this system has been adapted for RNA interference and gene editing in eukaryotic cells [3] as well as for detecting the presence of specific gene mutations for molecular diagnosis [4]. Cas13a is a newly discovered member of the Cas protein family that targets single-stranded RNA (ssRNA). In contrast to Cas9, which relies on tracrRNA and crRNA to recognize and cleave DNA, Cas13a only requires appropriate crRNA to recognize and cleave ssRNA. In addition, activated Cas13a exhibits collateral indiscriminate cleavage activity. When the HEPN domain within Cas13a is recognized and activated by crRNA, the Cas13a-crRNA-target RNA complex forms and specifically cleaves the target RNA, while nonspecific cleavage occurs in the surrounding RNAs [5]. Based on the biological feature of Cas13a, Gootenberg J S and colleagues established a novel nucleic acid detection system, named Specific High-Sensitivity Enzymatic Reporter Unlocking (SHERLOCK) that combines Cas13a with recombinase polymerase amplification (RPA) technology [5]. To date, the SHERLOCK system has been used extensively for the detection of SARS-CoV-2 [6, 7], Zika virus [8], Ebola virus [9], hepatitis B [10], *Mycobacterium tuberculosis* [11], *Staphylococcus aureus* [12], *Yersinia pestis* [13] and other pathogens [14, 15]. The system has demonstrated excellent sensitivity, specificity and portability, making it a rapid and quantitative testing method. However, the application of SHERLOCK to detect antibiotic resistance genes has not yet been widely implemented. In this study, we established a rapid RPA-Cas13a assay based on the SHERLOCK system for the *bla*_KPC_ gene that addresses several limitations of current methods for antibiotic resistant bacteria detection, such as heavy dependence on slow and laborious bacterial culture.

## Materials and Methods

### Materials and Instruments

#### Clinical Strains and Sputum Samples Collection

A total of 117 bacterial strains were isolated and collected from sputum samples of individual patients with suspected intestinal infection treated at our hospital during the first half of 2021. Carbapenemase production was confirmed by the Modified Carbapenem Inactivation Method (mCIM) and the presence of the *bla*_KPC_ gene was obtained by DNA sequencing. Among these isolated strains, 72/117 were identified as *Klebsiella pneumonia* (*KP*), 31/117 as *Escherichia coli* (*E. coli*), 2/117 as *Enterobacter aerogenes*, 4/117 as *Enterobacter cloacae*, 1/117 as *Proteus mirabilis*, 5/117 as *Pseudomonas aeruginosa*, and 2/117 as *Serratia marcescens*. In addition, 311 sputum samples were collected from individual patients treated at our hospital during the second half of 2021 for further clinical validation of the tests described in the study. All of the above samples were cultured and identified by mass spectrometry and antibiotic susceptibility tests. The standard strain *Klebsiella pneumoniae* ATCC BAA-1705 was used as a positive control while *Klebsiella pneumoniae* ATCC 700603 and *Escherichia coli* ATCC 25922 were used as negative controls. All standards were purchased from the American Type Culture Collection (ATCC, Manassas, VA, USA). The above samples were stored at −80 °C in our microbiology laboratory.

#### Reagents and Instruments

Primers for RPA and recombinant plasmid KPC-PC6813Gn/H5128-3 were ordered from Generay (Shanghai, China). In vitro transcribed (IVT) templates for crRNA, commercial LwaCas13a and FAM-UUUUUU-Quencher used as the fluorescent reporter were ordered from Bio-lifesci (Guangzhou, China). Hipure Bacteria DNA Kit for DNA extraction from Magen (Guangzhou, China), ApexHF HS DNA Polymerase FS Master Mix for PCR from Accurate Bio (Hunan, China) and the TwistAmp Basic Kit for RPA amplification from TwistDx (Cambridge, United Kingdom) were purchased. T7 RNA Polymerase Kit and RNase-free water were purchased from TaKaRa (Tokyo, Japan). RNase Inhibitor, the dNTP Mix (40 U/μL and 25 μM), Trypsin–EDTA (1X) and Agarose were purchased from Beyotime (Shanghai, China). Imipenem (IPM) and Meropenem (MEM) Susceptibility Test Disks (10 μg) were purchased from ThermoFisher Scientific (Waltham, USA), Blood agar plate and Mueller–Hinton agar (MHA) plate were purchased from Kailin Microbio (Jiangmen, China). The Trans 2 K DNA Marker Kit was purchased from TransGen Biotech (Beijing, China).

Bacterial strains were identified with VITEK MS and VITEK-2 Compact bacterial identification and antibiotic sensitivity system (Biomerieux, France). The PCR and RPA amplification were measured with ABI GeneAmp 9700 PCR System (ABI, United States). The amplification was confirmed by 1.2% agarose gel electrophoresis with Hema GSG-2000 gel imaging system (Zhuhai, China). The fluorescence quantifications were measured with CFX 96 Real-Time PCR System (Bio-Rad, United States). The DNA sequencing service was supported by Sangon Biotech Co. (Shanghai, China).

### Methods

#### Confirmation of Carbapenemase Production in Clinical Strains

The mCIM [16] was used to identify if the 117 clinical strains collected in "Clinical Strains and Sputum Samples Collection" section were capable of producing carbapenemase. Following the addition of 1 μL of the tested bacterial colony to 2 mL of tryptone soy broth (TSB), an antibiotic disk containing 10 μg Meropenem (MEM) was added and incubated at 37 °C for 4 h. In parallel, a suspension of mCIM indicator bacteria (*E. coli* ATCC 25922) at 0.5 McFarland turbidity was prepared and inoculated on MHA plates. Following that, the incubated disks were placed invertedly on MHA plates and incubated at 37 °C for 24 h. Finally, the diameter of the antibacterial zone around each MEM disk was measured as an indication of carbapenemase production. A positive result was defined as an antibacterial zone measuring between 6 and 15 mm in diameter or between 16 and 18 mm with scattered colonies present, indicating carbapenemase production by the tested bacteria. When the diameter measured between 16 and 18 mm or ≥ 19 mm with scattered colonies present, uncertainty regarding carbapenemase production by the tested bacteria arose. Conversely, a negative result indicated no carbapenemase production when the diameter measured ≥ 19 mm.

**Fig. 1:**
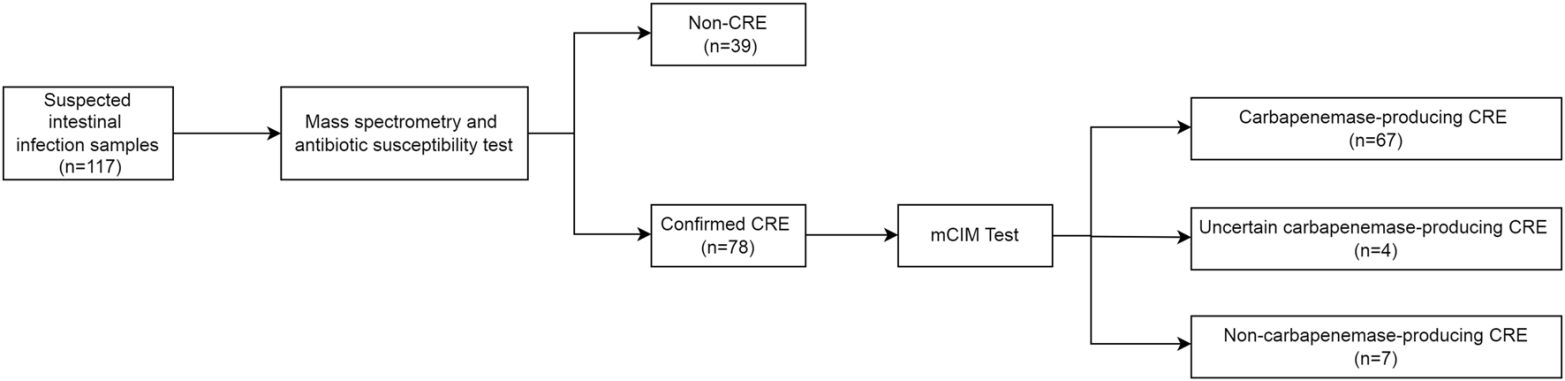
Flow chart showing the classification of 117 clinical samples with suspected intestinal infection

According to the results of mass spectrometry and the mCIM, 117 clinical strains were preliminarily divided into carbapenemase-producing CRE (Group A), uncertain carbapenemase-producing CRE (Group B), non-carbapenemase-producing CRE (Group C), and non-CRE (Group N) (Fig. 1).

#### Extraction of Genomic DNA

The frozen strains were recovered and cultured at 37 °C for 18–24 h on blood agar plates. A single bacterial colony (2–3 mm in diameter) was picked to prepare 1 McFarland bacterial suspensions (≈3 × 10^8^ CFU/mL). The bacterial genomic DNA was extracted from 117 strains and 311 sputum samples using the Hipure Bacteria DNA Kit from Magen, following the extractive steps: high-speed centrifugation at 12,000 rpm/min, digestion with lysozyme and water bath at 70 °C, repetitive silica gel column adsorption for purification to remove impurities, and subsequent elution of DNA. Sputum samples need to be pre-digested with trypsin before DNA extraction, getting the bacterial genomic DNA after the above extractive steps. The entire process for a single sample takes about one hour. The extracted DNA was stored at −80 °C.

#### Design and Optimization of Primers

The RPA primers for *bla*_KPC_ amplification were designed according to the methods described in previous studies [17, 18]. Briefly, to identify the highly conserved region of the *bla*_KPC_ gene, the complete coding DNA sequences of 166 members from the *bla*_KPC_ gene family available in NCBI were aligned using DNAMAN (GenBank accession numbers involved: AY034847.1, AF395881.1, EU447304.1, EU400222.2, EU555534.1, etc.). Primers were then designed using Primer Premier 5. The detailed primers and crRNA sequences are listed in Table 1.

**Table 1:** Sequences of RPA primers, KPC-crRNA, and RNA probe used to amplify and identify *bla*_KPC_

Name	Sequences (5′–3′)	Fragment length (bp)
Primer pair 1	KPC-F1: CACTGTGCAGCTCATTCAAGGGCTTTCT	210
	KPC-R1: atatttaatacgactcactatagggAATTGGCGGCGGCGTTATCACTG TATTG	
Primer pair 2	KPC-F2: GTTCTGCTGTCTTGTCTCTCAT	379
	KPC-R2: atatttaatacgactcactatagggCGGCGGCGTTATCACTGTATTG	
Primer pair 3	KPC-F3:gaaattaatacgactcactatagggCTTCAGCAACAAATTGGCGGCGG CGTTATC	222
	KPC-R3: CCACTGTGCAGCTCATTCAAGGGCTTTCTT	
KPC-crRNA	GAUUUAGACUACCCCAAAAACGAAGGGGACUAAAACUCACCCAUCUCGGAAAAAUAUCUGACAAC	–
RNA-probe	5′-FAM-UUUUUU-3′-BHQ1	–

The T7 promoter sequence is marked by lower-case; FAM is the fluorophore; BHQ1 is a quenching group

The purified DNA extracts from the positive standard strain, *Klebsiella pneumoniae* ATCC BAA-1705, were quantified using spectrophotometry (55.05 ng/μL). The extracts were then serially diluted from 1 to 1/1000 at a 1/10 ratio. Each diluted DNA templates were amplified separately by each of the three primer pairs (Table 1), visualized by gel electrophoresis and detected by the RPA-Cas13a assay synchronously. The primer pair that showed the highest yield of amplification products and the lowest yield of non-target products from non-specific amplification was chosen for further analysis of the clinical sample set.

#### Amplification of the *bla*_KPC_ Gene by PCR and RPA

The aforementioned DNA products and the KPC-PC6813Gn plasmid served as templates for *bla*_KPC_ amplification by PCR and RPA. The 20-μL PCR reaction system for *bla*_KPC_ amplification consisted of 6 μL RNase-free water, 10 μL ApexHF HS DNA Polymerase, 1 μL Primer KPC-F& KPC-R (10 μM), and 2 μL DNA template. The thermocycling conditions for the PCR reaction included an initial denaturation step at 98 °C for 10 min, followed by 40 cycles of denaturation at 98 °C for 10 s, annealing at 60 °C for 5 s, extension at 72 °C for 10 s, and a final extension step at 72 °C for 10 min. The PCR amplification products were subsequently confirmed through electrophoresis on a 1.2% agarose gel using the Hema GSG-2000 gel imaging system.

The 50-μL RPA reaction mixture was prepared following the instructions of the RPA TwistAmp Basic Kit, and consisted of 11 μL RNase-free water, 29.5 μL Primer Free Rehydration Buffer, 2.5 μL Primer KPC-F&KPC-R (10 μM), 2.5 μL MgOAc, and 2 μL DNA template. Based on the RPA amplification schemes summarized by Lobato' team [19] and the manufacturer's recommendations for RPA reaction conditions, we conducted the RPA reaction conditions at a constant temperature 39 °C for 30 min using the ABI Geneamp 9700 PCR system.

#### Detection of the *bla*_KPC_ Gene by the RPA-Cas13a Assay

The RPA-Cas13a assay for the *bla*_KPC_ gene was established according to previously successful protocols [11, 13]. The 20 μL reaction system was prepared as follows: RNase free water 11.7 μL, T7 RNA Polymerase 0.5 μL, 10 × T7 Buffer 2 μL, dNTP Mix (25 μM) 0.8 μL, Cas13a (1 μM) 1 μL, crRNA (1 μM) 0.5 μL, RNase Inhibitor (40 U/μL) 0.5 μL, FAM fluorescent probe (10 μM) 1 μL, and RPA products 2 μL. The reaction was conducted at 37 °C for 45 min on a Bio-Rad CFX 96 Real-Time PCR System. The relative fluorescence intensity (RFI) was recorded every minute.

#### Evaluation of the Lower Limit of Detection (LLD) and the Precision of the RPA-Cas13a Assay

The LLD is defined as the lowest concentration at which a signal is observed that exceeds the mean blank signal by three standard deviations, interpreted as a 99.7% probability that the sample contains the target analyte [20]. In this study, a positive detection signal was determined using the following formula: final ${\text{RFI}}>{\text{LLD}}= \overline{\text{X}}_{\text{Blank}} + 3 \times {\text{SD}}_{\text{Blank}}$. The blank control contained RNase-free water, and the final $\overline{\text{X}}_{\text{Blank}}\, \text{and} \,{\text{SD}}_{\text{Blank}}$ values were calculated from 4 replicates per day over 5 consecutive days by the RPA-Cas13a assay. The standard plasmid KPC-PC6813Gn was serially diluted ten-fold from 10^6^ copies/μL down to 1 copy/μL and each dilution tested 5 times by the RPA-Cas13a assay.

The standard plasmid KPC-PC6813Gn with 10^6^, 10^3^ and 10 copies were selected as samples from high, medium, and low concentrations for precision evaluation of the RPA-Cas13a assay. Each concentration was detected with double tubes and repeated 10 times to calculate the intra-assay coefficient of variation (Intra-CV). Similarly, each concentration was detected with double tubes once a day for 10 consecutive days to calculate the inter-assay coefficient of variation (Inter-CV). CV = X_final RFI_/SD_Final RFI_.

#### Construction and Evaluation of the RPA-Cas13a Assay Using Clinical Strains

**Fig. 2:**
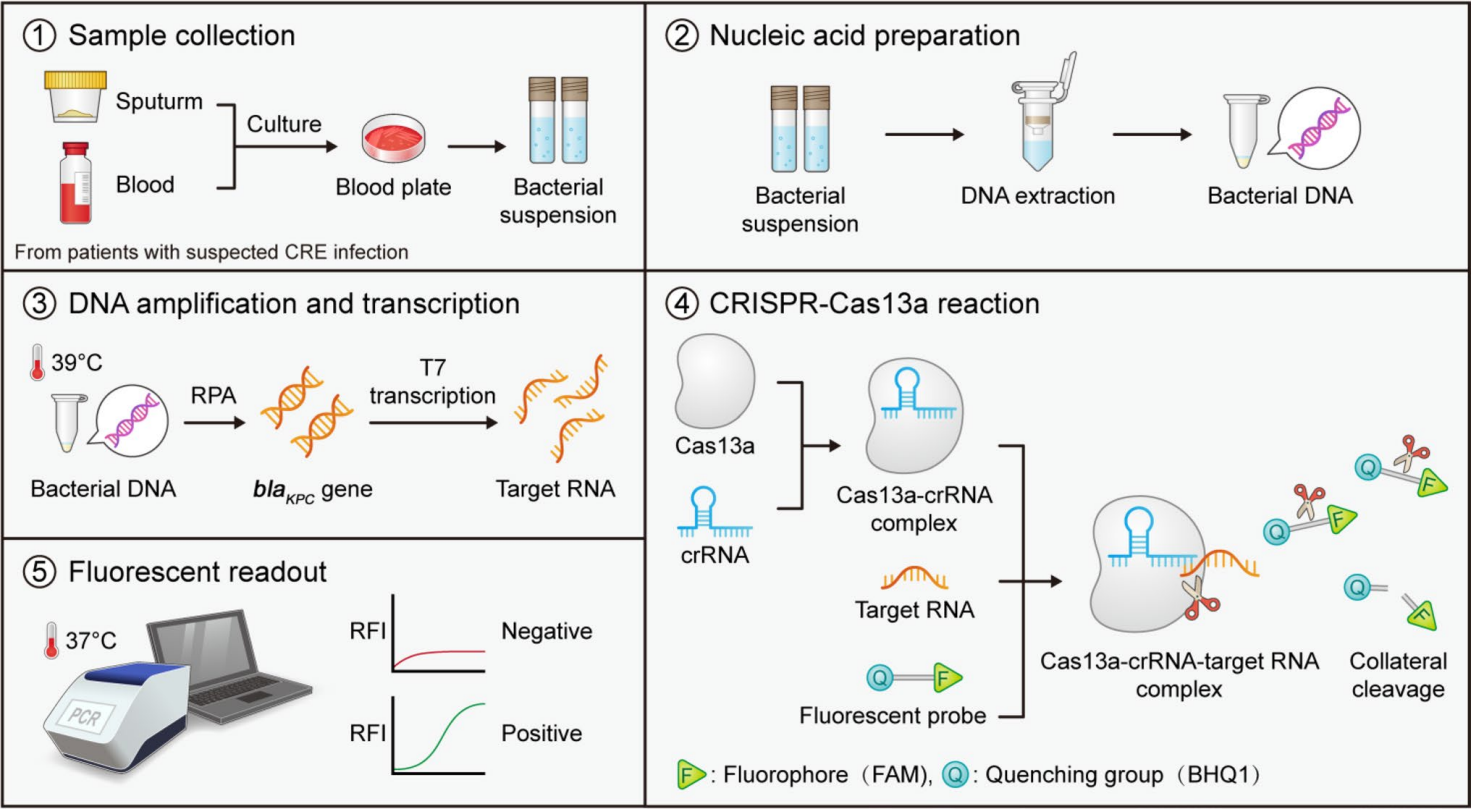
Working principle of the proposed RPA-Cas13a assay for *bla*_KPC_ detection

The working principle of the proposed method for *bla*_KPC_ detection is illustrated in Fig. 2. The RPA primers and crRNA targeting *bla*_KPC_ were designed based on NCBI sequences. Bacterial genomic DNA was extracted from suspensions raised from clinical strains and sputum samples of patients. The target RNA was transcribed from the *bla*_KPC_ gene amplified by RPA. In the CRISPR-Cas13a reaction, the Cas13a protein is activated by crRNA and binds to the target RNA forming a Cas13a─crRNA─target RNA complex, which leads to the cutting of the target RNA. Simultaneously, the collateral cleavage activity of Cas13a is also activated, resulting in the cutting of the surrounding FAM probe quenching molecules and enhancing the fluorescent signal emission.

DNA sequencing, as a gold standard, was applied to confirm the presence of the *bla*_KPC_ gene in clinical strains mentioned in "Clinical Strains and Sputum Samples Collection" section. The sensitivity of the RPA-Cas13a assay was defined as the ratio of positive rates detected by RPA-Cas13a and DNA sequencing for the *bla*_KPC_ gene detection, while the specificity was defined as the ratio of negative rates detected by both methods for the *bla*_KPC_ gene detection. Meanwhile, receiver operating characteristic (ROC) curve analysis was performed using data from 117 clinical strains to evaluate the diagnostic performance of RPA-Cas13a, with area under ROC curve (AUC) used as a measure.

#### Evaluation of the RPA-Cas13a Assay Using Clinical Sputum Samples

The *bla*_KPC_ gene status in 311 sputum samples was directly determined using the RPA-Cas13a assay. Simultaneously, these sputum samples underwent culture, mass spectrometry and antibiotic susceptibility testing. The results obtained from the RPA-Cas13a assay were compared to those of laboratory tests to assess their correlation and consistency.

### Statistical Analyses

All statistical analyses were performed using SPSS 22.0 and Graphpad Prism 9.0. Continuous variables were compared by one-way repeated measures ANOVA with post hoc Bonferroni correction for multiple comparisons. Rates were compared by the *Kappa* test. *P* < 0.05 was considered statistically significant for all tests.

## Results

### Identification and Classification of Clinical Strains

A total of 117 clinical strains were collected, including 78 carbapenem-resistant strains and 39 carbapenem-sensitive strains. Carbapenemase production was confirmed in these strains using mCIM and further divided into four groups (Fig. 1; Table 2). The mCIM screening showed that out of the 78 positive strains, 67 could produce carbapenemase (Group A), which included 51 *KP*, 13 *E.coli*, and one each of *E. aerogenes*, *E. cloacae*, and *P. aeruginosa*. Four carbapenem-resistant strains (3 KP and 1 *E. coli*) were classified as Group B due to reaching a critical antibacterial zone value where it couldn't be determined if they produced carbapenemase. Additionally, seven carbapenem-resistant strains with negative mCIM results were considered as CREs without carbapenemase production (Group C), while the remaining 39/117 carbapenem-sensitive strains belonged to Group N and also tested negative in mCIM.

**Table 2:** Identification and Classification of clinical strains

Bacteria	Groups				
	A	B	C	*N*	Total
*E. coli*	13	1	5	12	31
*E. aerogenes*	1	0	0	1	2
*E. cloacae*	1	0	0	3	4
*KP*	51	3	0	18	72
*P. mirabilis*	0	0	0	1	1
*P. aeruginosa*	1		2	2	5
*S. marcescens*	0	0	0	2	2
Total	67	4	7	39	117

### Optimization of Primer Pairs for *bla*_KPC_ Amplification

**Fig. 3:**
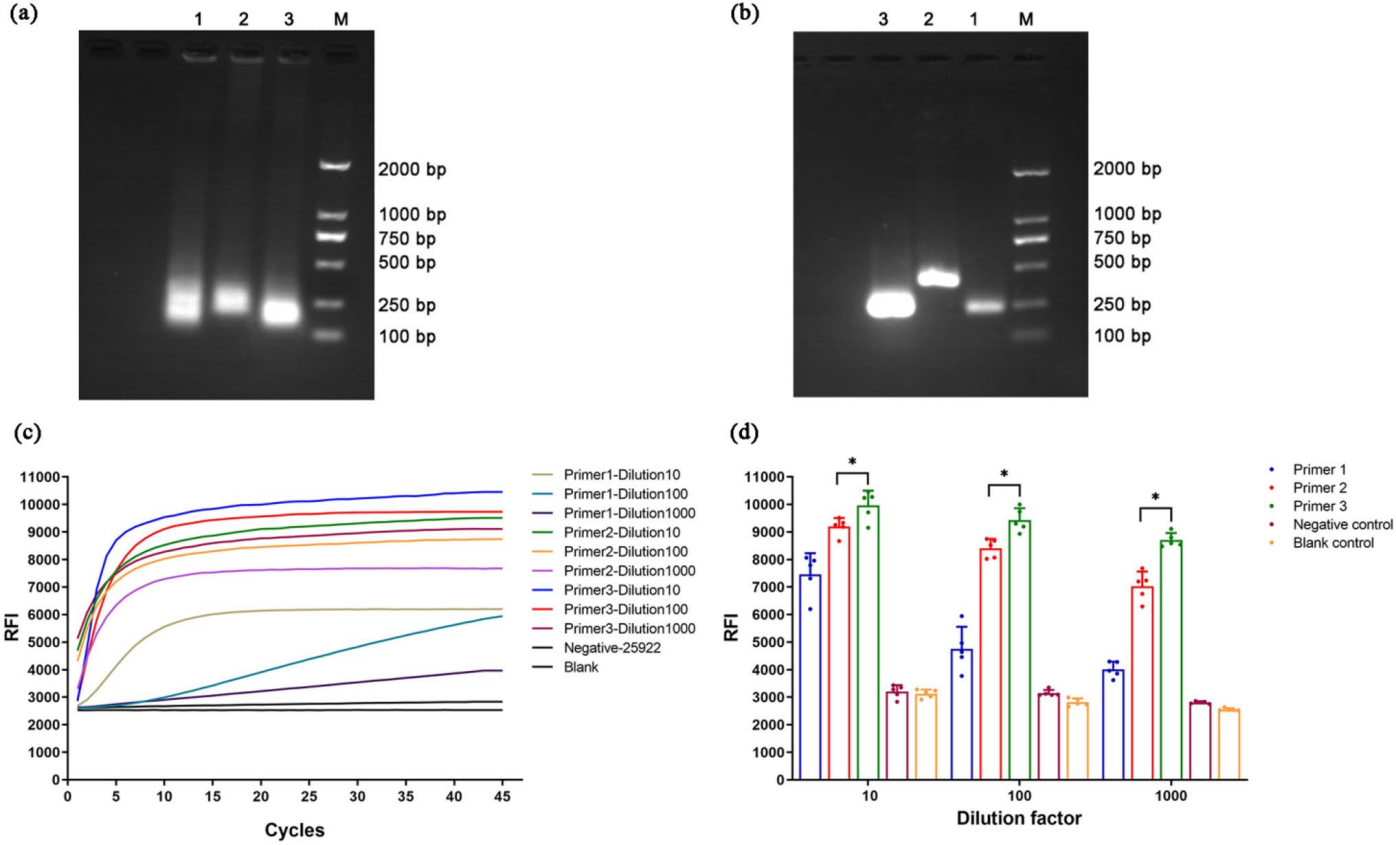
Comparison of *bla*_KPC_ amplification efficiency by RPA and PCR using primer pairs 1–3. **a** Agarose gel electrophoresis showing the RPA products obtained from standard strain ATCC BAA-1705 using each pair of primers (from left to right: primer pair 1, primer pair 2, primer pair 3, DNA Marker M 2 K). **b** Agarose gel electrophoresis showing the PCR products obtained using each primer pair (lane labels were indicated). **c** Real-time relative fluorescence intensity (RFI) curves during the *bla*_KPC_-targeted RPA-Cas13a assay of RPA products from ATCC BAA-1705 amplified at different dilutions (10-, 100-, and 1000-fold) using primer pairs 1–3. **d** The final RFI values for each condition. Data in (**d**) was presented as the mean ± SD. * P < 0.05 between the indicated groups. Based on amplification efficiency, prime pair 3 was selected for *bla*_KPC_ detection in clinical samples

All primer pairs successfully amplified the *bla*_KPC_ gene from the BAA-1705 standard strain in both PCR and RPA reactions. However, the electrophoretic bands following RPA were blurry (Fig. 3a). In addition, primer pair 1 demonstrated non-specific amplification in PCR, possibly due to the production of primer dimers (Fig. 3b). To determine the best overall primer pair, we performed separate RPA amplifications of tenfold serially diluted standard plasmid ATCC BAA-1705 using primer pairs 1, 2, and 3, and then compared the RFI values during RPA-Cas13a assay for the yielded RPA products. Notably, primer pair 3 consistently yielded significantly higher fluorescence signals across all three dilutions compared to primer pairs 1 and 2 (Fig. 3c, d). In fact, at a 1000-fold dilution, the final RFI signal generated by primer pair 1 was nearly undetectable while those produced by primer pair 2 were significantly lower than those achieved with primer pair 3 at both tenfold and 100-fold dilutions (Fig. 3d, both *P* < 0.05). Based on these findings, primer pair 3 was selected for further validation with clinical samples.

### Assesment of LLD and Precision of the RPA-Cas13a Assay

**Fig. 4:**
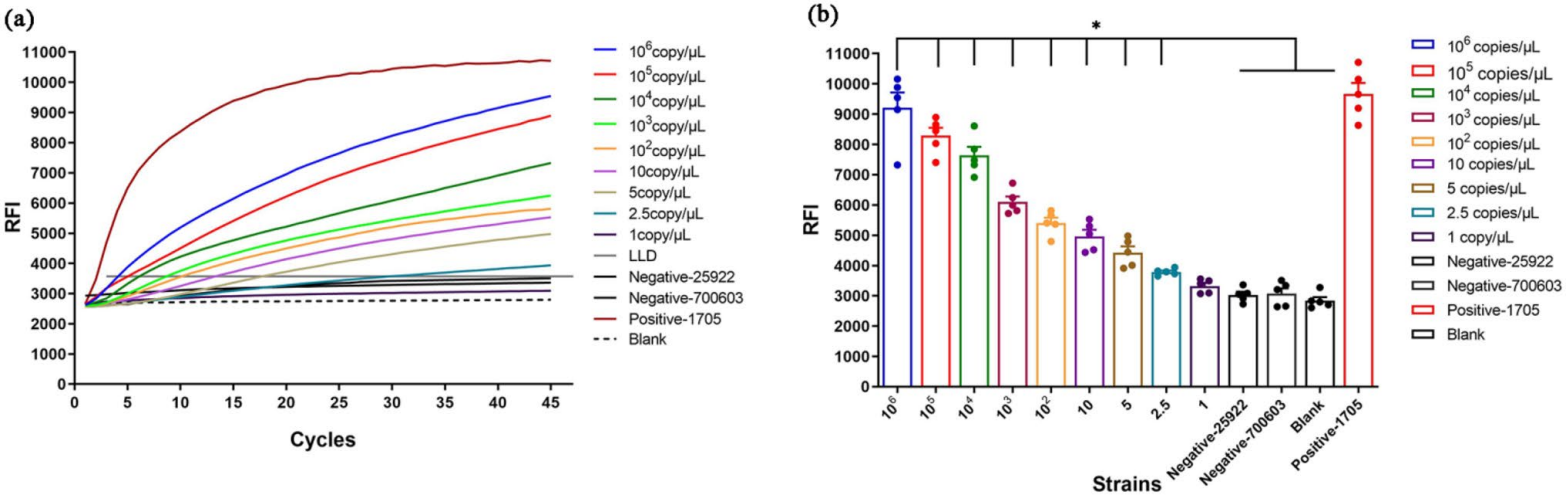
The LLD of the RPA-Cas13a assay for the *bla*_KPC_ gene. **a** Real time RFI curves showing the LLD of the RPA-Cas13a assay for the *bla*_KPC_ gene. **b** Final RFI values. Data in (**b**) are presented as the mean ± SD. *P < 0.05 compared to *bla*_KPC_ -negative and blank groups

There were no significant differences in the daily mean RFI of the blank control by the RPA-Cas13a assay across the 5 test days (*F* = 0.986, *P* > 0.05 by one-way repeated measures ANOVA; Table 3). The mean and SD for these blank control RFI values yielded an estimated LLD of ≈ 3600 according to the formula ${\text{LLD}}= \overline{\text{X}}_{\text{Blank}} + 3 \,{\text{SD}}_{\text{Blank}}\, (2972.62 + 3\, \times 200.43 \approx 3600).$ Therefore, fluorescence signal intensity above 3600 was considered dependent on the *bla*_KPC_ gene. We then performed the RPA-Cas13a assay on the standard plasmid KPC-PC6813Gn serially diluted from 10^6^ to 1 copy per μL to estimate the lowest copy number yielding an RFI value > 3600. According to these measurements, the RPA-Cas13a system could detect *bla*_KPC_ DNA fragments at a concentration of 2.5 copies/μL (Fig. 4).

**Table 3:** The final RFI values of blank control tested by the RPA-Cas13a assay across the 5 test days

Times	Days					
	1	2	3	4	5	20 times blank
1	3078.85	3094.56	3122.38	3352.52	3082.33	–
2	3278.90	2911.75	3164.87	3240.38	3026.19	–
3	2727.20	2924.68	2989.20	2770.05	2656.73	–
4	2809.88	2861.03	2832.61	2752.70	2775.71	–
${\overline{\text{M}}}\text{ean}$	2973.7075	2948.0050	3027.2650	3028.9125	2885.2400	2972.62
SD	252.85227	101.49138	149.80155	312.37982	202.42283	200.43

*F* = 0.986, *P* > 0.05

The coefficient of variation (CV%) was used to evaluate the precision of RPA-Cas13a assay. The intra-CV% of RPA-Cas13a when detecting recombinant plasmids with high, medium and low concentrations were 3.05, 4.29 and 4.92%, all of which were less than 5% (Table 4). The inter-CV% of RPA-Cas13a detection of high, medium, and low concentrations of recombinant plasmids were 6.38, 7.92, and 9.84%, all of which were less than 10% (Table 4).

**Table 4:** The precision of the RPA-Cas13a assay for *bla*_KPC_ gene

Concentration (copies/μL)	Mean of 10 times tests (final RFI)	SD of 10 times tests	Intra-CV (%)	Mean of 10 days tests (final RFI)	SD of 10 days tests	Inter-CV (%)
High (10^6^)	9697.68	296.24	3.05	9269.28	591.70	6.38
Medium (10^3^)	6529.56	280.31	4.29	6382.26	505.19	7.92
Low (10)	4916.76	242.10	4.92	4706.23	463.32	9.84

### Evaluation of the RPA-Cas13a Assay Using Clinical Strains

DNA sequencing, as the gold standard, was applied to identify the *bla*_KPC_ gene in all 117 clinical strains (Table 5; Fig. 6a, b). Among the 67 strains in Group A, all tested positive for the carbapenemase gene, with 54 strains carrying *bla*_KPC_ and 13 strains carrying *bla*_NDM_. Out of the 51 Carbapenem-resistant *Klebsiella pneumonia* (CRKP), 96.08% (49/51) were found to carry *bla*_KPC_ while 3.92% (2/51) carried *bla*_NDM_. Among the 13 Carbapenem-resistant *Escherichia coli* (CREC), 15.38% (2/13) carried *bla*_KPC_, 84.62% (11/13) carried *bla*_NDM_, and the remaining strains of *E. aerogenes*, *E. cloacae* and *P. aeruginosa* were all confirmed to carry *bla*_KPC_. None of the other strains in Group C and Group N tested positive for *bla*_KPC._ It is worth noting that three strains of *KP* in Group B tested positive for *bla*_KPC_, which contradicted their mCIM results.

**Table 5:** The results of 117 clinical strains identified by the mCIM, RPA-Cas13a assay and DNA sequencing

mCIM	Groups	Species of bacteria	RPA-Cas13a assay	DNA sequencing	Number of strains
(+)	Group A	*KP*	*bla*_KPC_ (+)	*bla*_KPC_ (+)	47
(+)	Group A	*KP*	*bla*_KPC_ (−)	*bla*_KPC_ (+)	2
(+)	Group A	*E.coli*	*bla*_KPC_ (+)	*bla*_KPC_ (+)	2
(+)	Group A	*E. aerogenes*	*bla*_KPC_ (+)	*bla*_KPC_ (+)	1
(+)	Group A	*E. cloacae*	*bla*_KPC_ (+)	*bla*_KPC_ (+)	1
(+)	Group A	*P. aeruginosa*	*bla*_KPC_ (+)	*bla*_KPC_ (+)	1
Critical	Group B	*KP*	*bla*_KPC_ (+)	*bla*_KPC_ (+)	3
(+)	Group A	*KP*	*bla*_KPC_ (−)	*bla*_NDM_ (+)	2
(+)	Group A	*E.coli*	*bla*_KPC_ (−)	*bla*_NDM_ (+)	11
Critical	Group B	*E.coli*	*bla*_KPC_ (−)	*bla*_KPC_, *bla*_NDM_ (−)	1
(−)	Group C	*E.coli*	*bla*_KPC_ (−)	*bla*_KPC_, *bla*_NDM_ (−)	5
(−)	Group C	*P. aeruginosa*	*bla*_KPC_ (−)	*bla*_KPC_, *bla*_NDM_ (−)	2
(−)	Group N	*E.coli*	*bla*_KPC_ (−)	*bla*_KPC_, *bla*_NDM_ (−)	12
(−)	Group N	*E. aerogenes*	*bla*_KPC_ (−)	*bla*_KPC_, *bla*_NDM_ (−)	1
(−)	Group N	*E. cloacae*	*bla*_KPC_ (−)	*bla*_KPC_, *bla*_NDM_ (−)	3
(−)	Group N	*KP*	*bla*_KPC_ (−)	*bla*_KPC_, *bla*_NDM_ (−)	18
(−)	Group N	*P. mirabilis*	*bla*_KPC_ (−)	*bla*_KPC_, *bla*_NDM_ (−)	1
(−)	Group N	*P. aeruginosa*	*bla*_KPC_ (−)	*bla*_KPC_, *bla*_NDM_ (−)	2
(−)	Group N	*S. marcescens*	*bla*_KPC_ (−)	*bla*_KPC_, *bla*_NDM_ (−)	2

**Fig. 5:**
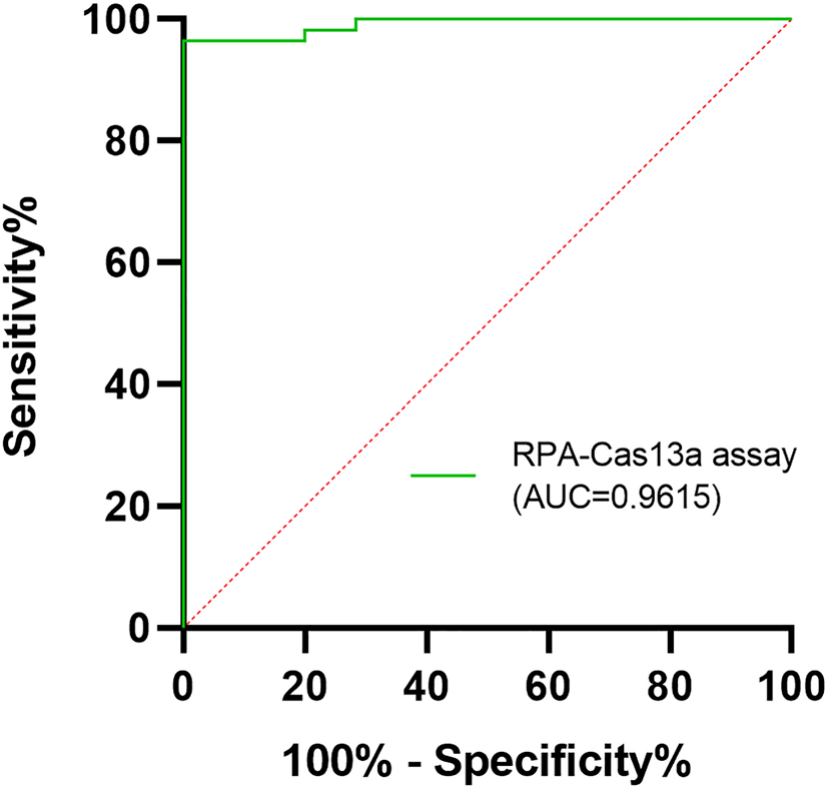
The ROC curve of the RPA-Cas13a assay for *bla*_KPC_ gene detection. The AUC was 0.9615,* P* < 0.01

**Fig. 6:**
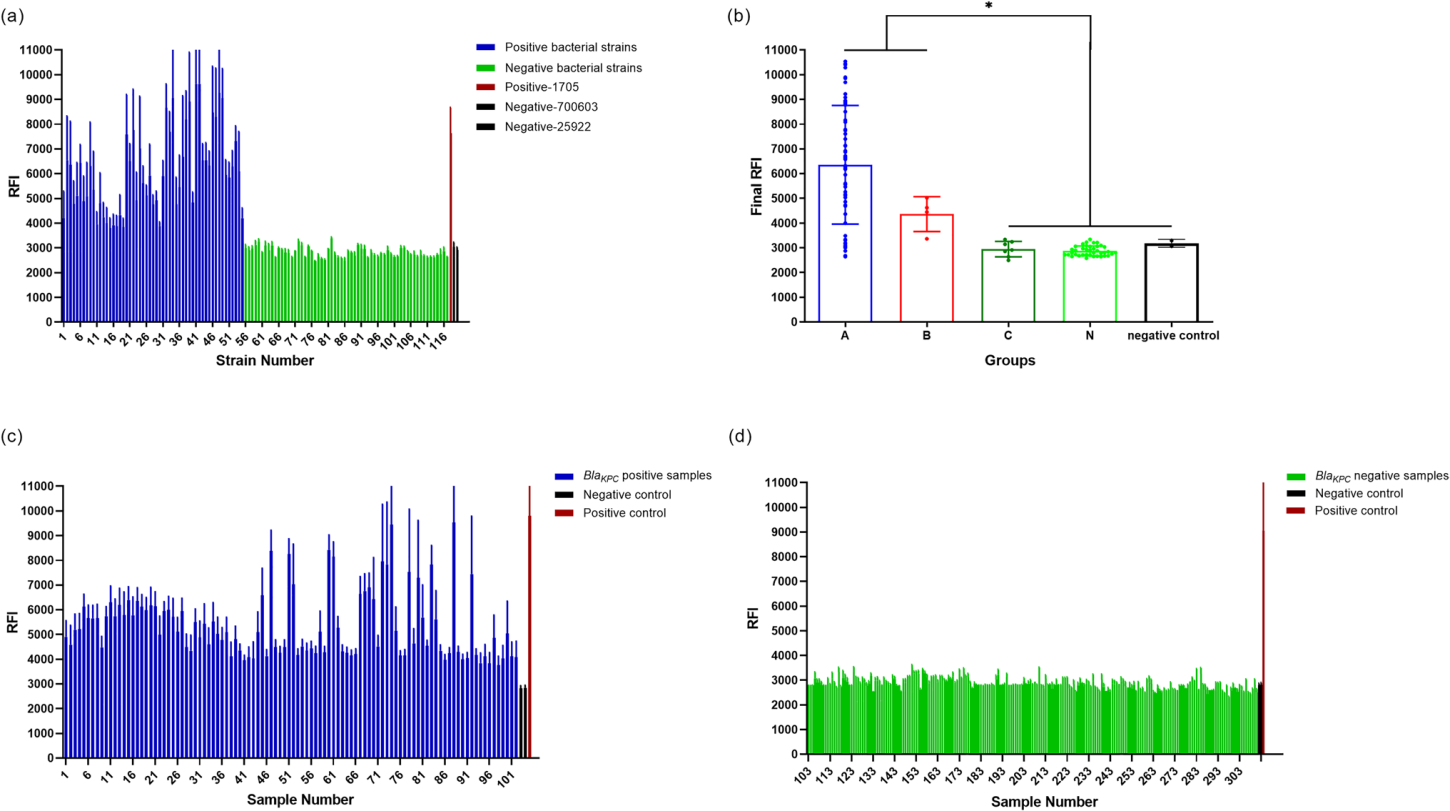
The RFI from the RPA-Cas13a assay of clinical strains and sputum samples. **a** The RFI signal of the RPA-Cas13a assay of 117 clinical strains and standards. **b** The final RFI. Data in (**b**) are presented as the mean ± SD. *P < 0.05. The mean RFI of Group A and Group B strains was above that of Group C, Group N and negative controls in (**a**). **c** The RFI signal of the RPA-Cas13a assay on 102 *bla*_KPC_-positive sputum samples and standards. **d** The RFI signal of the RPA-Cas13a assay on 209 *bla*_KPC_-negative sputum samples and standards

We further employed RPA-Cas13a to assess the presence of the *bla*_KPC_ gene in 117 clinical strains. Positive fluorescence signals were observed in 52/54 *bla*_KPC_-positive strains in Group A and all three *bla*_KPC_-positive strains in Group B. In contrast, negative fluorescence signals were detected in all *bla*_KPC_-negative strains in Group C and Group N during RPA-Cas13a detection (Table 5; Fig. 6b). In addition, the fluorescence signals emitted by *bla*_KPC_-positive strains in Group A and Group B exhibited significantly higher intensity compared to those of negative controls (Fig. 6b; *P* < 0.05). Conversely, there was no significant difference between the fluorescence signals produced by strains in Group C or Group N and those of negative controls (Fig. 6b; *P*  > 0.05). The results of RPA-Cas13a demonstrated a high level of concordance with those of DNA sequencing (*Kappa* = 0.957 > 0.81, *P* < 0.01). The sensitivity and specificity values for detecting the *bla*_KPC_ gene using the RPA-Cas13a assay on clinical strain samples were found to be approximately 96.5 and 100%, respectively (Table 6). ROC curve analysis revealed that RPA-Cas13a achieved an AUC of approximately 0.9615, which was extremely close to 1 (Fig. 5), indicating that RPA-Cas13a had excellent diagnostic performance for identifying *bla*_KPC_ gene.

**Table 6:** Comparison of *bla*_KPC_ detection in 117 clinical strains by RPA-Cas13a assay and DNA sequencing

RPA-Cas13a assay	DNA sequencing		
	Positive	Negative	Total
Positive	55	0	55
Negative	2	60	62
Total	57	60	117

Note: *Kappa* = 0.957 > 0.81, *P* < 0.01

SHERLOCK: sensitivity = 55 / 57 × 100% = 96.5%; Specificity = 60 / 60 × 100% = 100%

### Evaluation of the RPA-Cas13a Assay Using Clinical Sputum Samples

The results of 311 sputum samples detected by the RPA-Cas13a assay were further compared with those of subsequent culture, mass spectrometry and antibiotic susceptibility tests. The RPA-Cas13a assay successfully identified 102 samples as positive for *bla*_KPC_ gene, while the remaining 209 samples tested negative (Fig. 6c, d). Further analysis revealed that among the 105 sputum samples cultured with *Klebsiella pneumoniae* resistant to IPM or MEM, 102 samples were positive for the *bla*_KPC_ gene according to the RPA-Cas13a assay. Additionally, among the 209 RPA-Cas13a negative samples, 99 samples were identified as *Klebsiella pneumoniae*, 70 as *Escherichia coli*, and 9 as *P. aeruginosa*. All 209 isolates were found to be sensitive to IPM and MEM, and the remaining 31 *bla*_KPC_ -negative sputum specimens failed to culture pathogenic bacteria. In summary, the sputum samples with *bla*_KPC_ gene positive detected by RPA-Cas13a method were basically consistent with their supposed antibiotic resistance characteristics and antibiotic susceptibility results (Table 7, *Kappa* = 0.978 > 0.81, *P* < 0.01).

**Table 7:** Comparison of *bla*_KPC_ detection in 311 clinical sputum samples by the RPA-Cas13a assay and antibiotic susceptibility tests

RPA-Cas13a assay	Antibiotic susceptibility tests to IPM and MEM		
	Positive	Negative	Total
Positive	102	0	102
Negative	3	206	209
Total	105	206	311

A result resistant to either IPM or MEM was considered positive and a result sensitive to both IPM and MEM was considered negative in antibiotic susceptibility tests. *Kappa* = 0.978 > 0.81, *P* < 0.01

## Discussion

According to the annual reports of China Antimicrobial Resistance Surveillance System (CARSS), *CRKP* and *CREC* are the main forces of CRE, and their mortality rates have been alarmingly increasing over the past five years [21]. The production of carbapenemases is the most significant mechanism of resistance in CRE, with class A KPC enzymes being one of the most commonly isolated carbapenemases in China, primarily found in *CRKP*. KPC inactivates carbapenems through hydrolysis, resulting in resistance [22, 23]. Previous epidemiological investigations have indicated that infections caused by *CRKP* expressing *bla*_KPC*-2*_ or *bla*_KPC-3_ genes have significantly higher mortality rates compared to infections caused by strains expressing other carbapenemase genes [24, 25]. Therefore, prompt identification of specific carbapenemase type is urgently required for effective control measures against CRE outbreaks, containment of infection progression, and reduction in mortality.

For now, the common phenotypic methods used for detecting carbapenemases include the Carba NP test, mCIM, eCIM, and carbapenemase inhibitor enhancement test [26, 27]. These methods are effective in quickly and easily confirming the presence of carbapenemases, but they can not directly detect specific carbapenemase genes, such as the *bla*_KPC_ gene. In this study, we found that the mCIM method is generally reliable for confirming the carbapenemase production (Tables 3 and 4), but it still relies on the extremely time-consuming bacterial culture process. It is also worth mentioning that among the 4 strains in Group B that were not confirmed to produce carbapenemase by the mCIM, DNA sequencing revealed that 3 of KP strains carried the *bla*_KPC_ gene, indicating that there were false-negative results for carbapenemase detection by the mCIM. The inconsistencies between the mCIM and DNA sequencing results may be due to manual measurement errors and the interference of multiple antibiotic resistance factors. In addition, Fluorescence quantitative PCR (qPCR) and DNA sequencing are the most common genotyping methods for identifying carbapenemases. While these methods effectively reduce the detection time and provide specific carbapenemase genotyping results, their implementation is limited by the high cost of reagents and instruments as well as the need for trained technicians, which hinders applications outside primary medical institutions.

Taking advantage of the convenience of rapid isothermal amplification of RPA and the collateral cutting activity of Cas13a, a novel molecular detection method based on Cas13a combined with RPA was established for rapid detection of *bla*_KPC_, which addressing the limitations of previous detection methods. The method requires the design of one pair of RPA primers and one crRNA sequence, which further reduces the difficulty and cost of detection compared to other isothermal amplification technologies [19, 28]. During the primer validation phase ("Identification and Classification of clinical strains" section), some electrophoresis results indicated the possibility of non-specific amplification, which suggests potential protein contamination and primer dimer interference during DNA extraction, amplification, and/or detection (Fig. 3a, b). The RPA-Cas13a detection system utilizes the activation of Cas13a protein guided by crRNA to specifically activate the collateral cutting activity of Cas13a, resulting in the non-specific cutting of the FAM fluorescent probe quenching groups and enhanced fluorescence emission. The high specificity of Cas13a's recognition determines the high specificity of the RPA-Cas13a assay for *bla*_KPC_ detection. In 117 clinical strains, the *bla*_KPC_ detection by the RPA-Cas13a assay showed comparable diagnostic accuracy with DNA sequencing (*Kappa* = 0.957 > 0.81, *P* < 0.01), demonstrating 96.5% sensitivity and 100% specificity (Table 6). Notably, RPA-Cas13a could accurately identify 3 KPC-positive strains in Group B that could not be identified by the mCIM. The performance evaluation of the RPA-Cas13a assay revealed a LLD of 2.5 copies/μL, which is comparable to RT-qPCR. The ROC curve showed that the AUC of RPA-Cas13a was up to 0.9615. On the other hand, both the intra-CV (< 5%) and inter-CV (< 10%) of RPA-Cas13a met the laboratory requirements for the precision of molecular diagnostic methods [29], providing evidence of its reproducibility and reliability.

It is worth noting that when detecting sputum specimens, IPM and MEM antibiotic susceptibility tests were positive in three specimens, but RPA-Ca13a tests showed negative. Further analysis using mass spectrometry and DNA sequencing indicated that the antibiotic-resistant bacteria in these three sputum samples were *Klebsiella pneumoniae* with *bla*_NDM_ (+) and *bla*_KPC_ (−). This suggests that the resistance to IPM and MEM was primarily due to the presence of *bla*_NDM_, rather than *bla*_KPC_. It also highlights that the antibiotic susceptibility test can only observe the resistance phenotype of bacteria, while the RPA-Cas13a assay is able to identify the genotype associated with resistance. Apart from the above-mentioned, the theoretical antibiotic resistance characteristics of 102 sputum samples tested positive for *bla*_KPC_ gene by the RPA-Cas13a assay, found to be highly consistent with the results of mass spectrometry and antibiotic susceptibility tests. These findings indicate that the RPA-Cas13a assay demonstrates superior diagnostic efficiency in detecting *bla*_KPC_ gene.

Obviously, *bla*_KPC_-positive signals (RFI > 3600) were observed after 20 min of the Cas13a reaction and enter the reaction plateau in 30 min (Figs. 3c, 4a). Consequently, the whole process of RPA amplification, Cas13a reaction, and fluorescence signal detection can be completed in one hour, which is more rapid than PCR-gel electrophoresis or DNA sequencing. The RPA-Cas13a assay does not require expensive equipment or complex reaction conditions, making it suitable for application in primary medical units, field hospitals and on-site emergency clinics. The cost of a single RPA-Cas13a test for the *bla*_KPC_ gene was only about 8 dollars.

In conclusion, we successfully established a rapid, sensitive, and specific platform for *bla*_KPC_ detection from CRE using SHERLOCK. This assay can be used for rapid screening to initiate timely treatment and to monitor the distribution and spread of CRE in hospitals and communities. Moreover, our study demonstrates the feasibility of SHERLOCK for detecting antibiotic resistance genes. The visualization of fluorescence signals can be replaced by more convenient lateral flow immune-chromatographic strips, and multiple genes can be simultaneously detected in our future study.
